# Time-resolved visible and infrared absorption spectroscopy data obtained using photosystem I particles with non-native quinones incorporated into the A_1_ binding site

**DOI:** 10.1016/j.dib.2016.04.031

**Published:** 2016-04-20

**Authors:** Hiroki Makita, Gary Hastings

**Affiliations:** Department of Physics and Astronomy, Georgia State University, Atlanta, Georgia, United States

**Keywords:** Photosynthesis, Photosystem I, Quinone, Bioenergetics, Time-resolved, Electron transfer

## Abstract

Time-resolved visible and infrared absorption difference spectroscopy data at both 298 and 77 K were obtained using cyanobacterial *menB*^−^ mutant photosystem I particles with several non-native quinones incorporated into the A_1_ binding site. Data was obtained for photosystem I particles with phylloquinone (2-methyl-3-phytyl-1,4-naphthoquinone), 2-bromo-1,4-naphthoquinone, 2-chloro-1,4-naphthoquinone, 2-methyl-1,4-naphthoquinone, 2,3-dibromo-1,4-naphthoquinone, 2,3-dichloro-1,4-naphthoquinone, and 9,10-anthraquinone incorporated. Transient absorption data were obtained at 487 and 703 nm in the visible spectral range, and 1950–1100 cm^−1^ in the infrared region. Time constants obtained from fitting the time-resolved infrared and visible data are in good agreement. The measured time constants are crucial for the development of appropriate kinetic models that can describe electron transfer processes in photosystem I, “Modeling Electron Transfer in Photosystem I” Makita and Hastings (2016) [1].

## Specifications table

**Table:** 

Subject area	*Physics, Biology*
More specific subject area	*Photosynthesis, Photosystem I*
Type of data	*Table, graph*
How data was acquired	*Time-resolved visible absorption difference spectroscopy: LP920 laser flash photolysis spectrometer (Edinburgh Instruments, Livingston, UK), Time-resolved FTIR absorption difference spectroscopy: Bruker Vertex80 FTIR spectrometer (Bruker Optics Inc., Billerica, MA)*
Data format	*Analyzed*
Experimental factors	*Eight different quinones were incorporated into isolated PSI particles*
Experimental features	*Transient absorption changes of PSI with non-native quinones were measured at 298 and 77 K*
Data source location	*Atlanta, GA*
Data accessibility	*Within the Data in Brief article*

## Value of the data

• Demonstrates an effect of quinone substitution on photosystem I electron transfer kinetics, at both 298 and 77 K.
• Provides reaction time constants for various electron transfer processes in photosystem I (PSI) with different quinones incorporated into the A_1_ binding site.
• Data presented will be of value in developing and assessing new theoretical models of the (bio)energetics in PSI.

## 1 Data

**Fig. 1:**
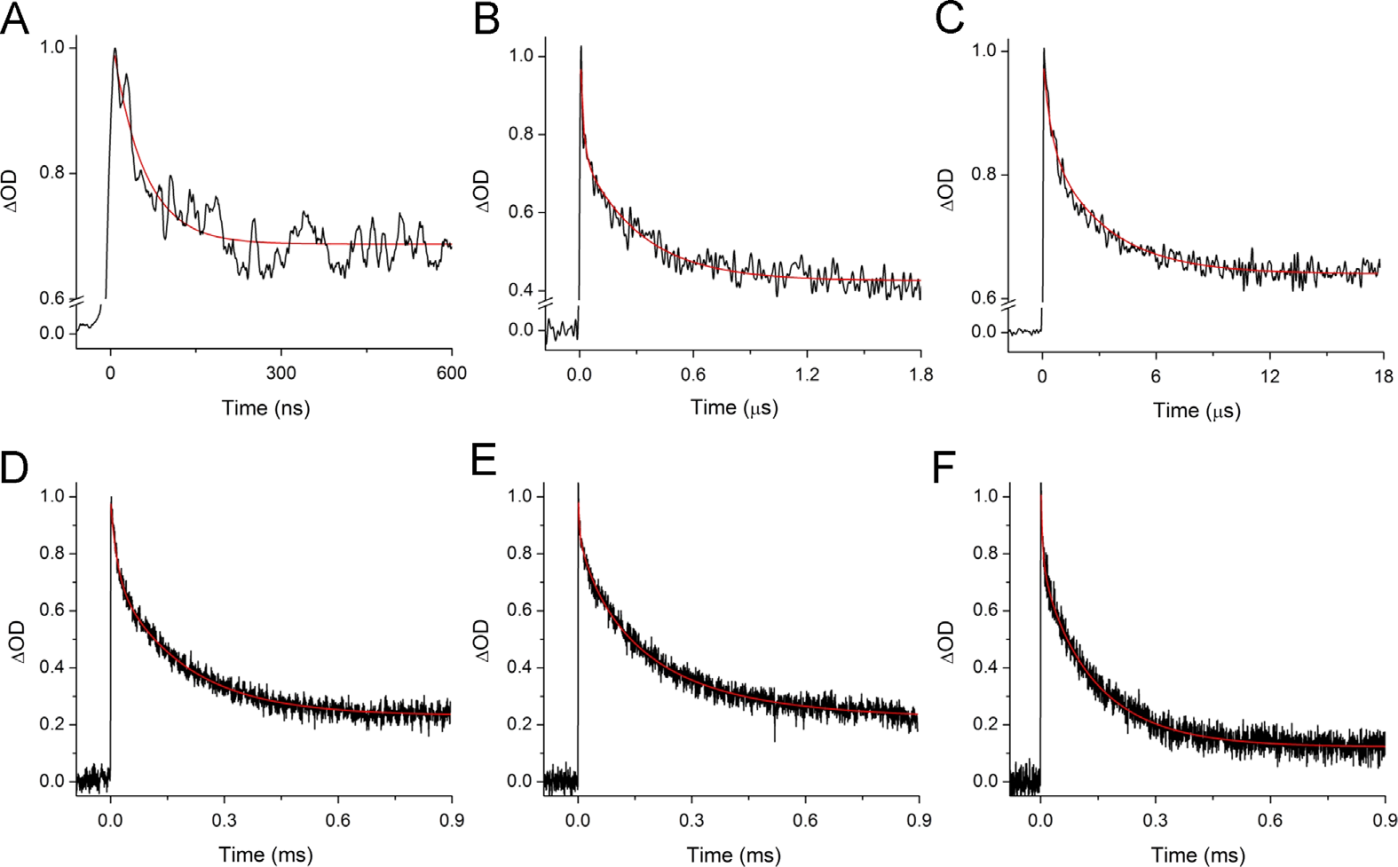
Room temperature (298 K) flash-induced absorption changes at 487 nm for PSI with (A) AQ, (B) PhQ, (C) 2MNQ, (D) 2ClNQ, (E) 2BrNQ, and (F) Cl_2_NQ incorporated into the A_1_ binding site. The data associated with forward ET (A–C) are fitted to a sum of exponential functions plus a constant. The data associated with charge recombination (D–F) are fitted to a sum of stretched exponential functions plus a constant. The fitted functions are also shown (red). The initial signal amplitudes were scaled. The timescales are selected to highlight the most prominent decay phases. The time constants obtained from fitting the data are listed on Table 1. The time constants of minor phase associated with P700^+^A_1_^−^ charge recombination (see reference [4]) are not included.

Fig. 1 shows room temperature (298 K) flash-induced absorption changes at 487 nm for PSI with 9,10-anthraquinone (AQ), phylloquinone (PhQ), 2-methyl-1,4-naphthoquinone (2MNQ), plastoquinone-9 (PQ_9_), 2-chloro-1,4-naphthoquinone (2ClNQ), 2-bromo-1,4-naphthoquinone (2BrNQ), and 2,3-dichloro-1,4-naphthoquinone (Cl_2_NQ) incorporated into the A_1_ binding site.

**Fig. 2:**
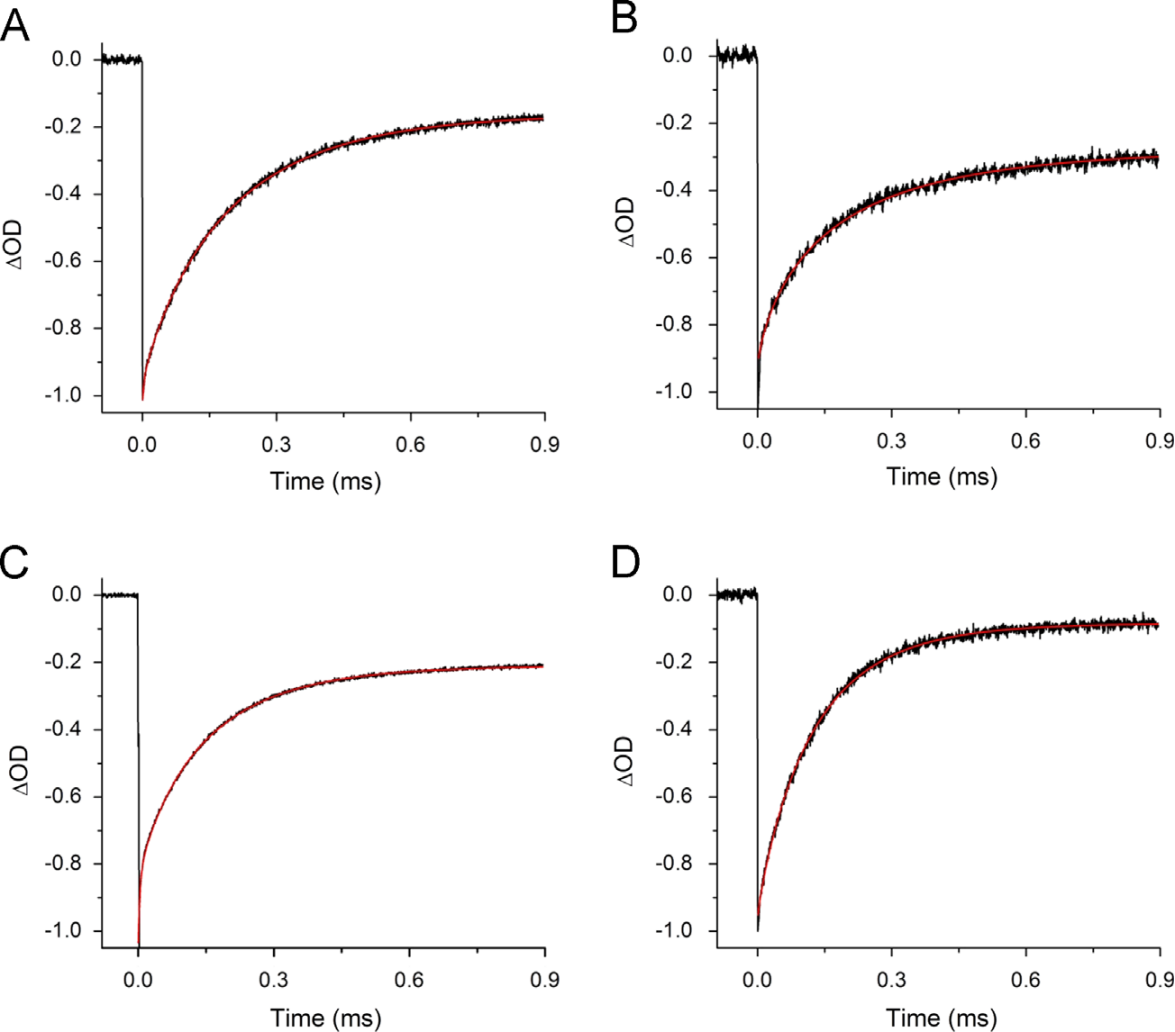
298 K flash-induced absorption changes at 703 nm for PSI with (A) 2ClNQ, (B) 2BrNQ, (C) Cl_2_NQ, and (D) Br_2_NQ incorporated into the A_1_ binding site. The data are fitted to a stretched exponential function plus a constant. The fitted functions are also shown (red). The initial signal amplitudes have been scaled. The time constant obtained from fitting the data are listed on Table 1.

Fig. 2 shows the 298 K flash-induced absorption changes at 703 nm for PSI with 2ClNQ, 2BrNQ, Cl_2_NQ, and Br_2_NQ incorporated. Similar data and conclusions follow from time-resolved infrared spectroscopy data (Fig. 4).

**Fig. 3:**
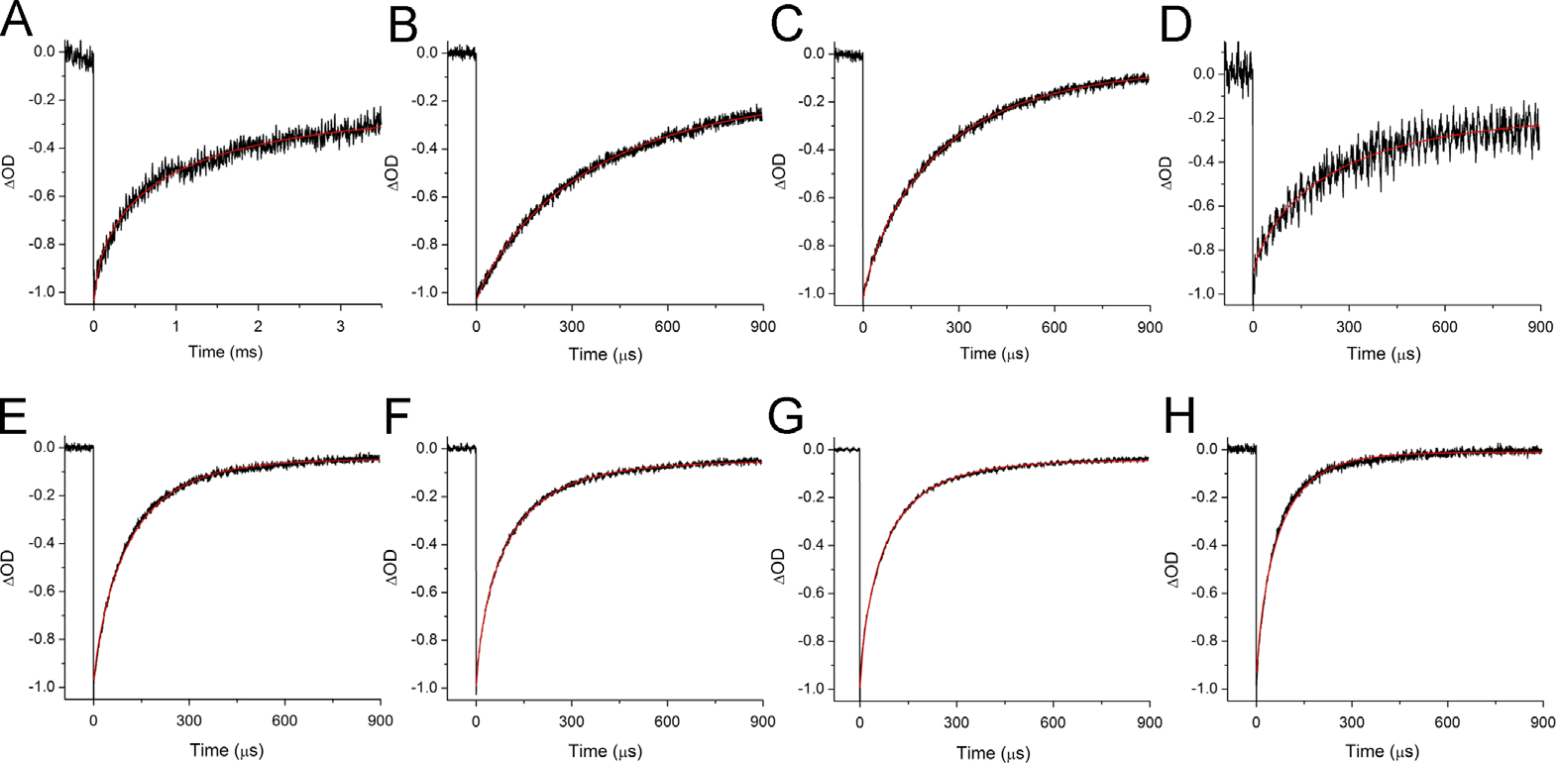
77 K flash-induced absorption changes at 703 nm for PSI with (A) AQ, (B) PhQ, (C) 2MNQ, (D) PQ_9_, (E) 2ClNQ, (F) 2BrNQ, (G) Cl_2_NQ and (H) Br_2_NQ incorporated. Fitted functions (stretched exponential plus a constant) are shown (red) and the calculated time constants are listed in Table 1.

Fig. 3 shows 77 K flash-induced absorption changes at 703 nm for PSI with AQ, PhQ, 2MNQ, PQ_9_, 2ClNQ, 2BrNQ, Cl_2_NQ and Br_2_NQ incorporated.

**Fig. 4:**
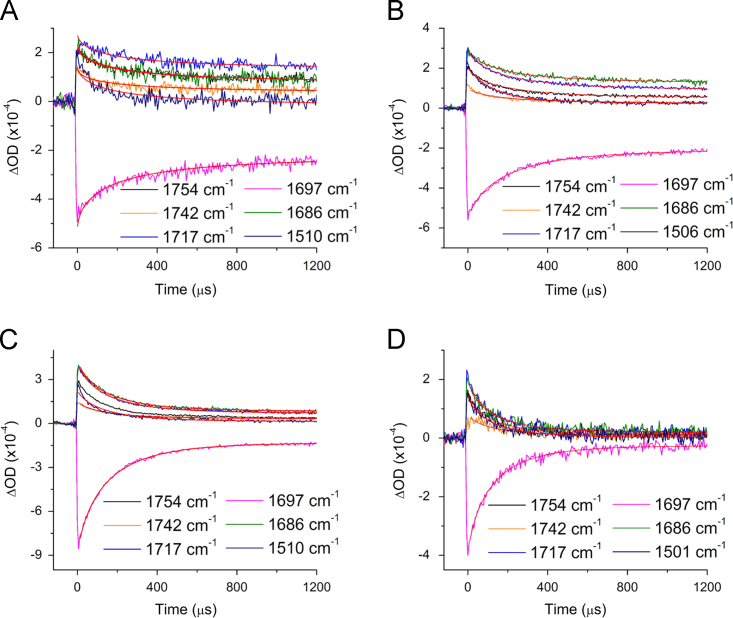
298 K time-resolved absorption changes at several infrared wavelengths (wavenumbers) for PSI with (A) 2ClNQ, (B) 2BrNQ, (C) Cl_2_NQ, and (D) Br_2_NQ incorporated. In each caption the shown wavelengths were fitted simultaneously to a stretched exponential function and a constant (red). The calculated time constants are listed in Table 1.

Fig. 4 shows time-resolved step-scan FTIR difference spectra obtained at 298 K for PSI with the high potential quinones 2ClNQ, 2BrNQ, Cl_2_NQ, and Br_2_NQ incorporated.

Table 1 summarizes the time constants obtained from fitting both the visible and infrared spectroscopic data at both 298 and 77 K.

## 2 Experimental design, materials and methods

Trimeric PSI particles from *menB*^−^ mutant cells from S6803 were isolated and stored as described previously [2]. All chemicals, including the series of quinones (AQ, PhQ, 2MNQ, ClNQ, BrNQ, Cl_2_NQ, and Br_2_NQ) incorporated into the A_1_ binding site, were obtained from Sigma-Aldrich (St. Louis, MO) and were used as received. To incorporate non-native quinones in to the A_1_ binding site, quinones dissolved in either ethanol or dimethyl sulfoxide were added to a suspension of *menB*^−^ PSI particles at ~500× molar excess. Concentrations of ethanol or dimethyl sulfoxide were kept below 2% of the total volume. The mixture was incubated at 277 K in the dark with continuous stirring for 24 h. The incubated mixture was pelleted by ultracentrifugation (408,000 g for 3 h). Sodium ascorbate (20 mM) and phenazine methosulfate (10 μM) were added to the pelleted mixture for rapid reduction of P700^+^. For a preparation of the concentrated thin-film samples, the pelleted samples were squeezed between two windows as described previously [3]. For a preparation of standard dilute samples, the pelleted samples were re-suspended in Tris buffer (pH 8.0) with 0.04% *n*-dodecyl-*β*-D-maltoside in a 1 cm path-length spectroscopic cuvette as described previously [3]. All the samples were prepared free of cryoprotectants. The concentrated thin-film samples were measured at 703 nm at 298 K and 77 K. The standard dilute samples were measured at 703 nm and 487 nm at 298 K. For measurements at 77 K, the samples were mounted in a Model ND1110H liquid nitrogen cooled cryostat (Cryo Industries of America In., Manchester, NH).

Nanosecond to millisecond time-resolved visible absorption difference spectroscopy was undertaken using an LP920 flash photolysis spectrometer (Edinburgh Instruments, Livingston, UK) as described previously [3], [4]. A Minilite or Surelite III Nd:YAG laser operating at 10 Hz repetition rate (Continuum, San Jose, CA) was used to provide 5–7 ns saturating pump pulses at 532 nm. Pump pulse intensity at the sample was ~2.6 mJ/cm^2^ (1 mJ pulses with spot diameter 0.7 cm at the sample). A pulsed xenon arc lamp was used as probe light source. A 1 cm water cell was placed between the probe light source and the sample to reduce heating effects on the sample. The probe wavelength was selected using a monochromator (Bentham Instruments TMc 300) placed between the sample and the detector. The probe light was detected using a Hamamatsu R928 photomultiplier tube. Interference filters (10 nm FWHM) were placed in front of the sample to reduce probe light actinic effects. Optical filters were also placed in front of the entrance slit of monochromator to attenuate scattered photons from the laser pump beam.

Microsecond time-resolved step-scan FTIR absorption difference spectroscopy was undertaken using a Bruker Vertex80 (Bruker Optics Inc., Billerica, MA) FTIR spectrometer, as described previously [3], [4]. The same laser excitation sources were used in both the infrared and visible absorption difference spectroscopy measurements. Data were collected in the 1950–1100 cm^−1^ region at 4 cm^−1^ spectral resolution. 2000–1000 cm^−1^ bandpass filters were placed between the IR light source and the sample, and between the sample and the detector. All samples were prepared on 1-inch calcium fluoride windows.

Flash-induced absorption changes are fitted to exponential or stretched exponential functions using the Levenberg–Marquardt algorithm implemented within Origin 7.5 (OriginLab Corporation, Northampton, MA).

## Figures and Tables

**Table 1:** Time constants obtained from fitting the experimental data in Fig. 1, Fig. 2, Fig. 3, Fig. 4, for PSI with eight different quinones incorporated into the A_1_ binding site, at 298 and 77 K. Q refers to the species of the incorporated quinone. ^a–d^ From Refs [5]^a^, [3]^b^*,*[6]^c^, and [7]^d^.

Q	298 K			77 K
487 nm	703 nm	IR	703 nm
AQ	50 ns	>100 ms^*c*^		797 µs
PhQ	25 ns^*a*^/310 ns	50–100 ms^*d*^		366 µs
2MNQ	430 ns/3.1 µs	14.4 ms^*b*^		239 µs
PQ9	13.9 µs^*b*^/202 µs^*b*^	3.2 ms^*b*^		250 µs
2ClNQ	188 µs	187 µs	185 µs	114 µs
2BrNQ	162 µs	165 µs	181 µs	94 µs
Cl_2_NQ	140 µs	140 µs	137 µs	78 µs
Br_2_NQ		124 µs	117 µs	70 µs
